# Genetic diversity analysis of Inner Mongolia cashmere goats (Erlangshan subtype) based on whole genome re-sequencing

**DOI:** 10.1186/s12864-024-10485-x

**Published:** 2024-07-16

**Authors:** Ruijun Wang, Xinle Wang, Yunpeng Qi, Yanbo Li, Qin Na, Huiping Yuan, Youjun Rong, Xiaofang Ao, Furong Guo, Lifei Zhang, Yan Liu, Fangzheng Shang, Yanjun Zhang, Yu Wang

**Affiliations:** 1 College of Animal Science, Inner Mongolia Agricultural University, Hohhot, 010018 China; 2 Inner Mongolia Autonomous Region Agricultural and Animal Husbandry Technology Extension Center, Hohhot, 010010 China; 3 Bayannur Forestry and Grassland Career Development Center, Bayannur, 015006 China; 4 College of Vocational and Technical, Inner Mongolia Agricultural University, Baotou, 014109 China; 5 Key Laboratory of Mutton Sheep Genetics and Breeding, Ministry of Agriculture, Hohhot, 010018 China; 6 Key Laboratory of Goat and Sheep Genetics, Breeding and Reproduction, Inner Mongolia Autonomous Region, Hohhot, 010018 China; 7 Northern Agriculture and Livestock Husbandry Technology Innovation Center, Hohhot, 010018 China; 8 College of Veterinary Medicine, Inner Mongolia Agricultural University, Hohhot, 010018 China

**Keywords:** Inner Mongolia cashmere goats, Genetic diversity, Kinship, Family structure, Inbreeding coefficient

## Abstract

**Background:**

Inner Mongolia cashmere goat (IMCG), renowned for its superior cashmere quality, is a Chinese indigenous goat breed that has been developed through natural and artificial selection over a long period. However, recently, the genetic resources of IMCGs have been significantly threatened by the introduction of cosmopolitan goat breeds and the absence of adequate breed protection systems.

**Results:**

In order to assess the conservation effectiveness of IMCGs and efficiently preserve and utilize the purebred germplasm resources, this study analyzed the genetic diversity, kinship, family structure, and inbreeding of IMCGs utilizing resequencing data from 225 randomly selected individuals analyzed using the Plink (v.1.90), GCTA (v.1.94.1), and R (v.4.2.1) software. A total of 12,700,178 high-quality SNPs were selected through quality control from 34,248,064 SNP sites obtained from 225 individuals. The average minor allele frequency (MAF), polymorphic information content (PIC), and Shannon information index (SHI) were 0.253, 0.284, and 0.530, respectively. The average observed heterozygosity (Ho) and the average expected heterozygosity (He) were 0.355 and 0.351, respectively. The analysis of the identity by state distance matrix and genomic relationship matrix has shown that most individuals’ genetic distance and genetic relationship are far away, and the inbreeding coefficient is low. The family structure analysis identified 10 families among the 23 rams. A total of 14,109 runs of homozygosity (ROH) were identified in the 225 individuals, with an average ROH length of 1014.547 kb. The average inbreeding coefficient, calculated from ROH, was 0.026 for the overall population and 0.027 specifically among the 23 rams, indicating a low level of inbreeding within the conserved population.

**Conclusions:**

The IMCGs exhibited moderate polymorphism and a low level of kinship with inbreeding occurring among a limited number of individuals. Simultaneously, it is necessary to prevent the loss of bloodline to guarantee the perpetuation of the IMCGs’ germplasm resources.

**Supplementary Information:**

The online version contains supplementary material available at 10.1186/s12864-024-10485-x.

## Background

China boasts an extensive array of indigenous cashmere goat breeds, chief among them being the renowned Inner Mongolian cashmere goat (IMCG), which encompasses several distinctive subtypes. The Erlangshan subtype is celebrated for its exceptionally fine cashmere fibers, the Albas subtype is distinguished by its remarkable productivity, and the Alashan subtype is acclaimed for its resilience and extraordinary ability to thrive in harsh environmental conditions [1]. Beyond the IMCGs, China is also home to the Liaoning, Hexi, Hanshan, Uzhumqin, Shaanxi, and Yanshan cashmere goats, each contributing unique qualities to the nation’s renowned cashmere industry [2]. Collectively, these diverse breeds have positioned China as a leading global force in cashmere production. China is the largest producer of cashmere globally, accounting for over 70% of the total global production, with approximately 30% attributed to IMCGs [3]. In 2021, China produced 15,102.18 tons of cashmere, representing over two-thirds of the worldwide output. IMCGs are an exceptional indigenous breed renown for producing both high-quality meat and exceptional cashmere, thus constituting a crucial cashmere goat genetic resource in China [4, 5]. IMCGs are well-known for remarkable traits such as outstanding drought and cold resistance, disease resistance, and a strong tolerance to coarse sustenance [6]. The cashmere from IMCGs is renowned for its fineness, softness, white luster, and high yield, earning it the nicknames “fiber gem” and “soft gold” [7, 8]. However, the genetic resources of IMCGs have been significantly threatened by the introduction of other goat breeds (Mongolian cashmere goats, Altai goats, Liaoning cashmere goats, Uzhumqin cashmere goats, Hanshan cashmere goats, and Hexi cashmere goats) and the absence of effective breed protection systems [6]. While the current research focuses on the selection and breeding of cashmere traits, differential gene expression, and molecular regulatory mechanisms of hair follicle development during the cashmere growth cycle in IMCGs, relatively little attention has been paid to preserving their genetic resources. This oversight may have neglected the issue of genetic resource loss within IMCGs, posing a significant challenge and threat to their genetic diversity. Consequently, the urgent priority is to conserve the genetic resources of IMCGs.

Genetic diversity assessment is a crucial aspect of livestock population conservation, necessitating a thorough comprehension of the genetic diversity and population structure of specific livestock breeds [9]. Advances in molecular biology have enabled the identification of numerous single nucleotide polymorphism (SNP) markers, which are abundantly distributed throughout the genome. These markers have been extensively utilized in genetic diversity studies of animal populations [10]. Various indicators, including minor allele frequency (MAF), polymorphic information content (PIC), Shannon information index (SHI), effective number of alleles (Ne), fixation index (Fi), observed heterozygosity (Ho), and expected heterozygosity (He), have been emphasized in population genetic diversity assessments [11, 12]. Furthermore, phylogenetic trees have emerged as an effective tool for elucidating population structure, while the analysis of runs of homozygosity (ROH) provides profound insights into inbreeding levels within populations [13, 14].

SNPs are a common type of genetic variation present throughout the genome. They are extensively utilized for various research purposes, including the evaluation of germplasm resources, analysis of genetic diversity, and the study of phylogenetic evolution [15–17]. Current SNP typing methods commonly include whole genome sequencing (WGS), genotyping by sequencing (GBS), and commercial SNP chips. Chen et al. [18] employed WGS to analyze Yunling cattle and confirmed that the breed maintained low genetic diversity during the selection process due to inbreeding. Tao et al. [19] analyzed the genetic diversity and structure of Tarim and Junggar Bactrian Camels in China using GBS. The results indicated that both types of Bactrian Camels exhibit rich genetic diversity and a close genetic relationship, with evident historical genetic exchange between them. The 50K SNP chip was utilized to assess genetic diversity and relationships within the Punjab goat breeds of Pakistan, revealing rich genetic diversity but a high degree of inbreeding within this population [20]. Whole genome re-sequencing (WGRS) involves high-throughput sequencing of individuals from species with known reference genome species, followed by the study of genetic differences among individuals through analysis of the measured genome data and comparison with the reference sequence [21, 22]. To evaluate the conservation effectiveness of IMCGs and to ensure effective preservation and utilization of the purebred germplasm resource, this study analyzed the genetic diversity, kinship, family structure, and inbreeding of IMCGs based on resequencing data of 225 randomly selected individuals. The objective of this study is to provide enhanced theoretical support and strategies for the preservation of the genetic resources of the IMCGs (Erlangshan subtype).

## Results

### Sequencing reads quality control, reads mapping, and SNPs calling

This study analyzed the genetic diversity, kinship, family structure, and inbreeding of IMCGs utilizing WGRS data from 225 randomly selected individuals analyzed using the Plink (v.1.90), GCTA (v.1.94.1), and R (v.4.2.1) software. Based on WGRS data, the average sequencing depth is 20X. The conservation population of IMCGs displayed moderate polymorphism. The majority of individuals within this conserved population are genetically distant from each other, with only a few exhibiting close kinship. First, we performed a quality control comparison of the sequencing data. The cleaned sequencing reads were mapped to the goat reference genome (ARS1, GCF_001704415.1) using BWA (v0.7.17) software. From the 225 individuals analyzed, a total of 34,248,064 SNPs were identified (Table 1). The quality control of the SNP data in IMCGs was presented in Table 1. Following the application of filtering conditions, such as MAF < 0.1, HWE *p* ≤ 10^–6^, and SNPs with call rates < 0.9 [23]. A total of 127,001,178 high-quality SNPs were obtained from 225 individuals. A comparison of the number of SNPs on each chromosome before and after quality control was illustrated in Figure S1, revealing significant variations in the SNP counts across different chromosomes. Notably, the physical locations of SNP sites on each chromosome were evenly distributed after quality control (Figure S2). When considering the physical length of each chromosome, it was shown that the coverage of SNP loci in this sequencing result was more comprehensive and the quality control conditions were reasonable.

**Table 1:** Statistics on SNPs quality control results

Quality control standards	Number of SNPs	Remaining SNPs
Total numbers of SNPs before quality control		34,248,064
SNPs with MAF < 0.10	20,888,831	13,359,233
HWE (*p* < 10^− 6^)	599,109	12,760,124
SNPs with call rate < 0.90	59,946	12,700,178
Total numbers of SNPs after quality control		12,700,178

*Notes* MAF, minor allele frequency; HWE, Hardy–Weinberg equilibrium. Calculated from the resequencing results of 225 randomly selected Inner Mongolian cashmere goat samples (Erlangshan subtype)

### Genetic diversity analysis

**Fig. 1:**
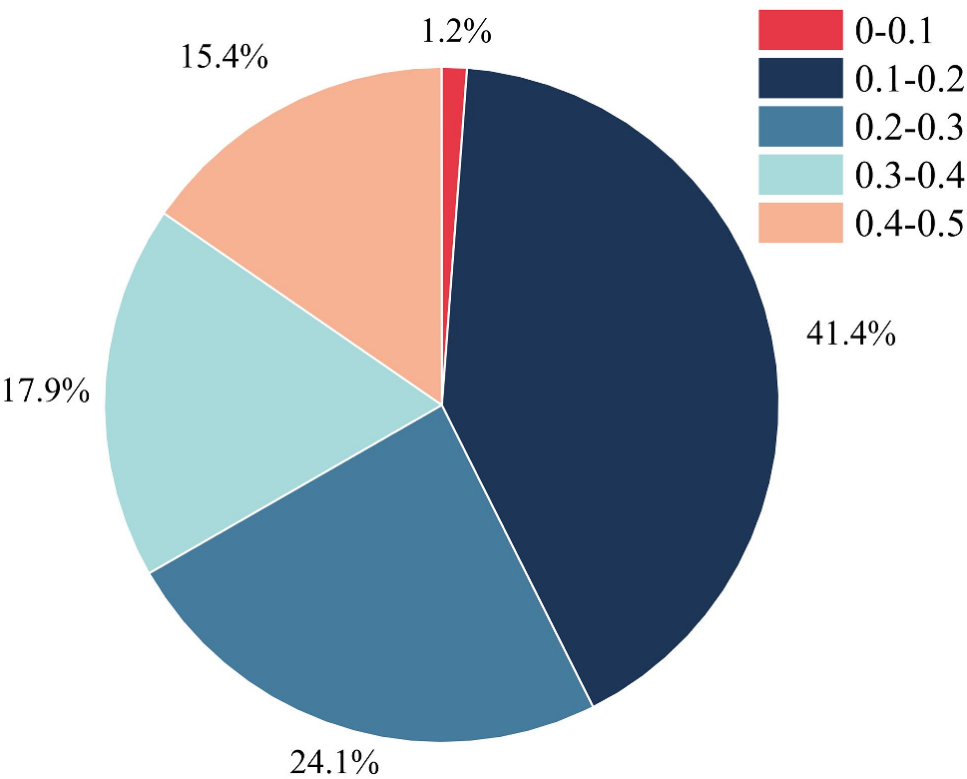
Distribution of minor allele frequency (MAF) for SNP after QC

**Fig. 2:**
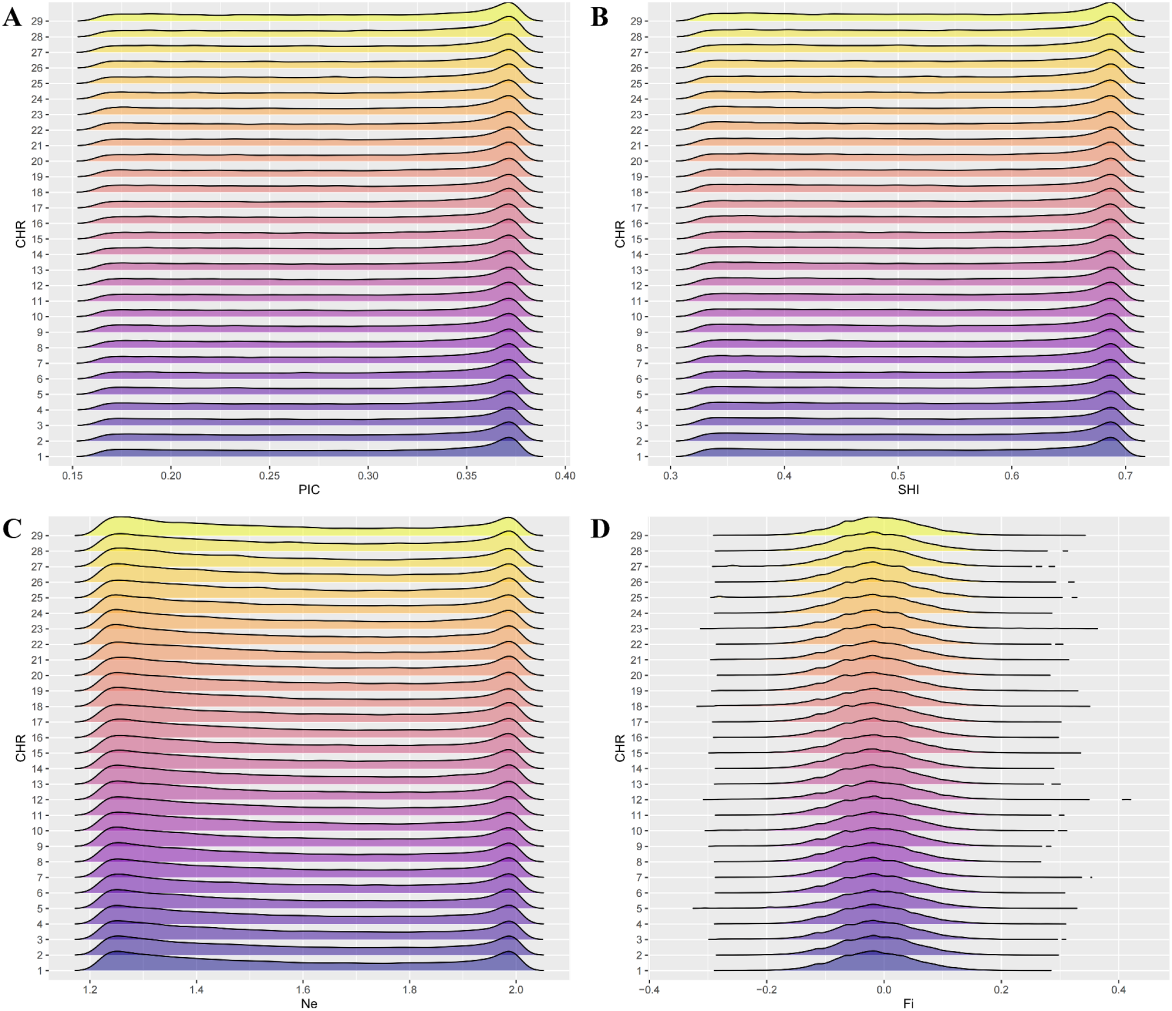
Distribution of different indicators of genetic diversity on chromosomes

The population genetic diversity parameters of IMCGs are summarized in Table 2. MAF values ranged from 0.100 to 0.500, averaging 0.253, with a concentration between 0.1 and 0.2 on the chromosomes (Fig. 1). PIC values ranged from 0.164 to 0.377, averaging 0.284, and were primarily concentrated between 0.35 and 0.40 on the chromosomes (Fig. 2A; Table 2). SHI values ranged from 0.325 to 0.693, averaging 0.530. The distribution pattern of SHI on various chromosomes primarily concentrated between 0.65 and 0.70 (Fig. 2B; Table 2). Ne values ranged from 1.220 to 2.004, averaging 1.582 (Fig. 2C; Table 2). The Fi values ranged from − 0.347 to 0.431, averaging − 0.013, and were concentrated between − 0.1 and 0.1 on the chromosomes (Fig. 2D). The genetic diversity results showed that the IMCGs were moderately polymorphic.

**Table 2:** Genetic diversity parameters of IMCGs (Erlangshan subtype) population

Population genetic diversity parameter	Numeric value
Minor allele frequency (MAF)	0.253
Polymorphism information content (PIC)	0.284
Shannon information index (SHI)	0.530
Effective number of alleles (Ne)	1.582
Fix- index (Fi)	-0.013
Observed heterozygosity (Ho)	0.355
Expected heterozygosity (He)	0.351

### Kinship analysis

**Fig. 3:**
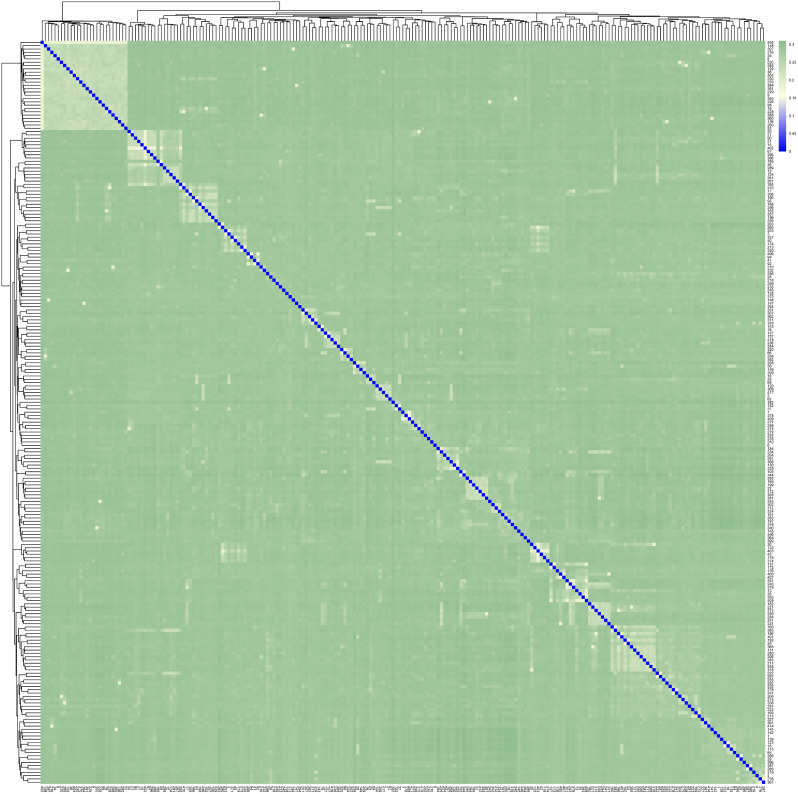
The Identity by state (IBS) distance matrix of IMCGs conserved population. Each small square in the figure represents the genetic distance value between the two pairs from the first individual to the last individual. The larger the value (the closer it is to green), the larger the genetic distance between two individuals, meaning they were not extremely similar, and vice versa

To investigate the kinship relationships within the conserved population of IMCGs, this study assessed the IBS genetic distance among 225 individuals throughout the whole IMCG population using Plink v1.90 software. The IBS matrix serves as a metric to quantify the similarity between two individuals within a population, based on their matching genotypic patterns at specific loci. Visualization of these results was achieved using the R programming language and is presented in Fig. 3. The IBS values ranged from 0.171 to 0.310, with an overall average genetic distance of 0.283. These findings indicate that the majority of individuals within the IMCGs population exhibit significant genetic distance, indicating a significant variation between the individuals. Only a few individuals demonstrated closer genetic similarities. Additionally, the study focused on the analysis of IBS genetic distances among 23 ram goats within the IMCGs. The results revealed that the IBS values for these 23 rams ranged from 0.173 to 0.302, with an average genetic distance of 0.280.

**Fig. 4:**
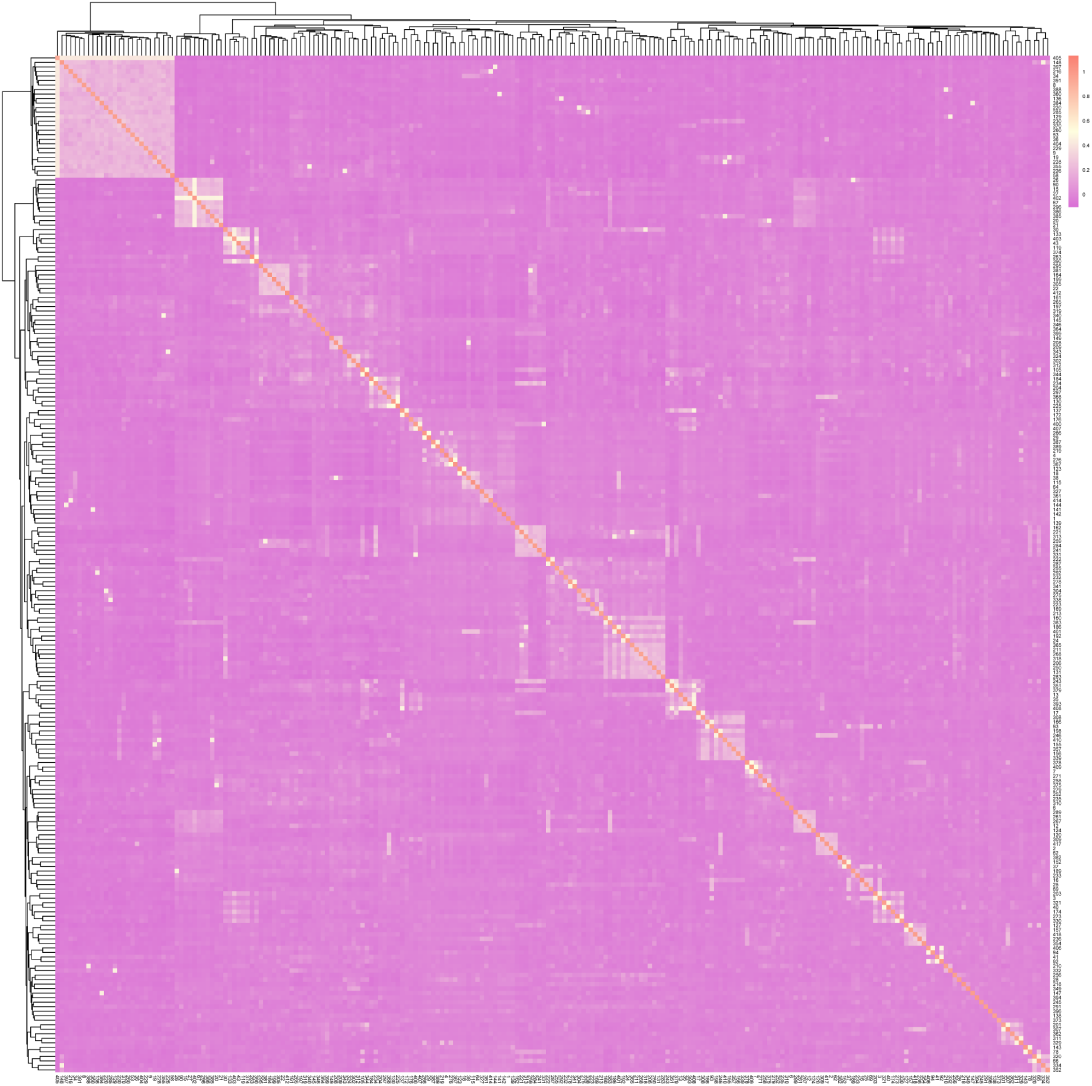
The G relationship matrix of IMCGs conserved population. Each small square in 230 the figure represents the relationship value between the two pairs from the first individual 231 to the last individual. The smaller the value (the closer it is to purple), the more distant the relationship between two individuals, and vice versa

The G-matrix offers a more precise measure of individual kinship through genome-wide SNP markers analysis, as compared to genealogy analysis alone. In practice, genealogical information of conserved populations is frequently inaccurate or incomplete, necessitating the use of the G-matrix to rectify recorded genealogical data. Following the post-quality control data, a kinship G-matrix was established for individuals within the IMCGs. The results are presented in Fig. 4, align with the IBS distance matrix. Among 225 individuals, a total of 25,200 kinship pairs were identified, resulting in an average kinship coefficient of -0.004. Notably, 72.25% of individuals exhibited kinship coefficients below 0, indicating substantial genetic distance. Conversely, 23.45% of individuals displayed kinship coefficients ranging from 0 to 0.1, suggestive of closer genetic proximity. The remaining 4.3% of individuals exhibited kinship coefficients exceeding 0.1, indicating a potential risk of inbreeding within the conservation population of the IMCGs (Erlangshan subtype). The results could be related to the putative level of relative among animals.

### Family structure analysis

**Fig. 5:**
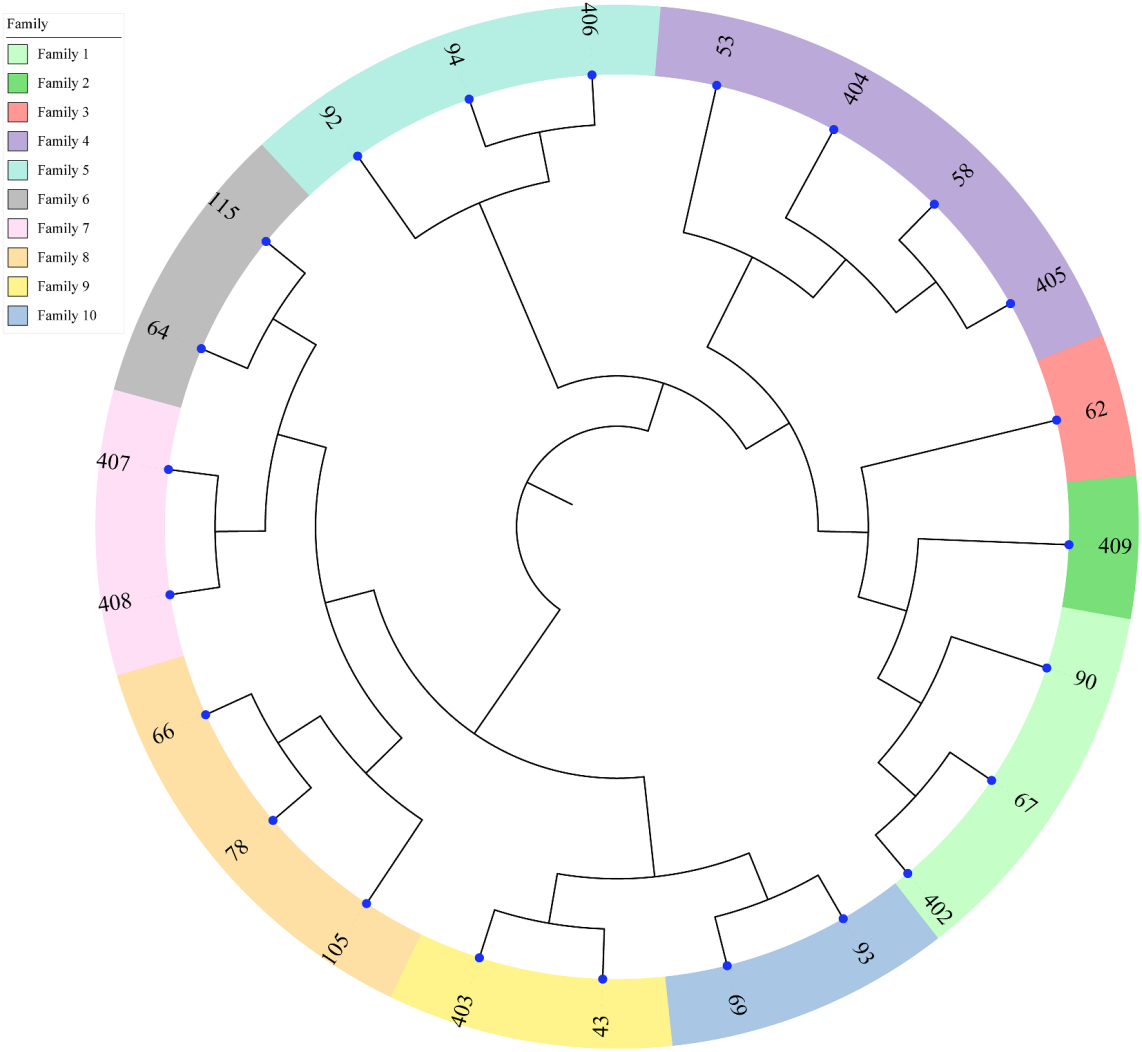
A neighbor-joining tree for IMCG rams

Given the significance of rams in the IMCG populations, we utilized MEGA (v10.0) to construct a rooted neighbor-joining (NJ) tree using the Maximum Likelihood evolutionary distance approach. This approach facilitated the delineation of the family structure among the 23 breeding rams. Using a criterion of a coefficient of relatedness exceeding 0.1 between rams, we categorized the 23 breeding rams into 10 distinct family lines. These families were numbered 1–10, and the results are presented in Fig. 5. Family No.4 comprises four breeding rams, while families No.1, No.5, and No.8 each contain three breeding rams. On the other hand, families No.6, No.7, No.9, and No.10 each have two breeding rams. However, families No.2 and No.3 are at risk of extinction as they are represented by a single ram each, thereby threatening the continuation of their bloodline.

### Detection of runs of homozygosity and genomic inbreeding coefficient

**Fig. 6:**
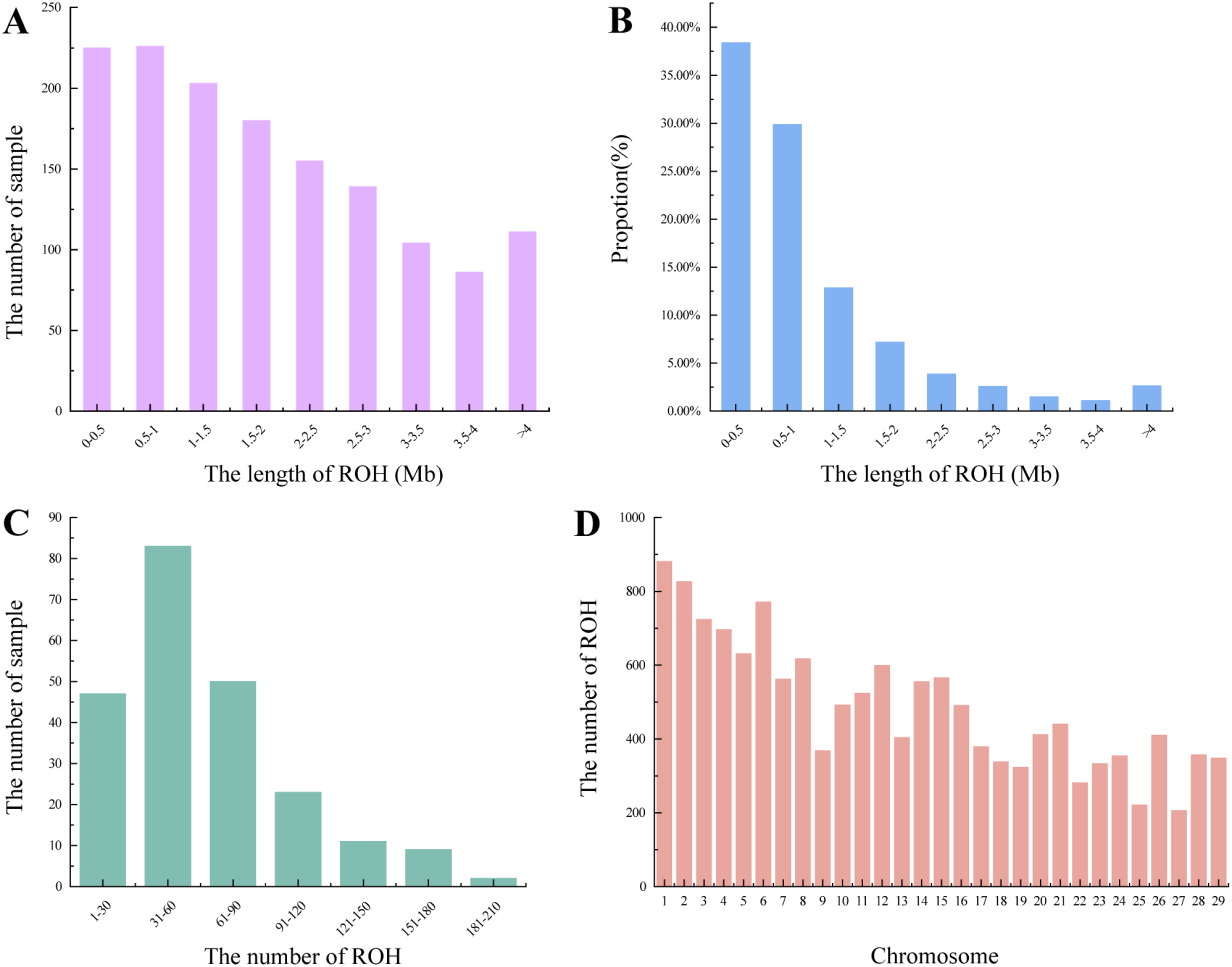
Distribution of ROH in IMCGs (Erlang subtype) population. (**A**) Sample number for different length categories (Mb) of ROH in IMCGs. (**B**) The average percentage for in different length categories (Mb) of ROH in IMCGs. (**C**) Sample number for different number categories of ROH in IMCGs. (**D**) Number distribution of ROH on each chromosome in IMCGs

A total of 14,109 ROHs were identified in 225 individuals of IMCGs, averaging 62.7 ROHs per individual. Each ROH averaged a length of 1014.547 kb, totaling 14.31 GB in length. 96.75% of the ROHs were homozygote, while only 3.00% were heterozygous. The shortest ROH was observed on chromosome 25, with a length of 101.466 kb, whereas the longest ROH was located on chromosome 17, with a length of 14,801.134 kb (Table 3). To gain a deeper understanding of the population history, the ROH was categorized into segments based on their physical lengths. Segments with ROH lengths of 0-0.5 Mb and 0.5-1 Mb were distributed among 225 individuals, with a decreasing number of individuals distributed as the ROH length increased (Fig. 6A). The highest number of ROHs, totaling 5174 fragments, fell within the lengths of 0-0.5 Mb, representing 38.39% of the total ROH count (Fig. 6B). Furthermore, significant variation was observed in the number of ROHs detected among individuals within the entire population, ranging from a minimum of 10 to a maximum of 212. 83 individuals were found to have 31–60 ROHs, representing 36.89% of the population examined. Additionally, 47 individuals had 1–30 ROHs, while 50 individuals had 61–90 ROHs (Fig. 6C). Chromosome 1 exhibited the highest number of ROHs, while chromosome 27 had the lowest (Fig. 6D).

**Table 3:** Statistics of ROH on chromosomes in IMCGs (Erlang subtype) population

Chr	Individuals number	ROH number	ROH length range (kb)	SNPs number	Percentage of ho	Percentage of he
1	208	881	120.948 ∼ 11140.410	247,537	96.47%	3.25%
2	174	826	149.799 ∼ 12967.640	275,110	96.69%	3.07%
3	165	724	107.623 ∼ 8518.012	214,504	96.74%	3.06%
4	180	696	101.934 ∼ 8873.662	220,427	96.75%	3.02%
5	170	631	104.852 ∼ 6111.188	187,417	96.44%	3.32%
6	189	771	132.000 ∼ 9336.397	229,504	96.76%	2.99%
7	165	562	125.377 ∼ 10163.166	191,232	96.57%	3.12%
8	148	617	178.591 ∼ 5884.146	192,846	96.88%	2.89%
9	139	368	113.496 ∼ 11766.695	133,780	96.87%	2.91%
10	147	492	113.100 ∼ 8966.650	159,889	96.60%	3.21%
11	159	524	167.135 ∼ 9066.409	143,281	96.63%	3.14%
12	174	599	109.212 ∼ 10445.348	184,433	96.82%	2.89%
13	145	404	140.045 ∼ 8581.239	127,052	96.61%	3.19%
14	170	555	120.566 ∼ 14300.658	165,476	96.61%	3.16%
15	165	566	140.488 ∼ 10110.782	166,784	96.73%	3.00%
16	149	491	168.147 ∼ 7516.709	153,726	96.79%	2.99%
17	136	379	118.177 ∼ 14801.134	114,614	96.80%	2.95%
18	117	338	123.470 ∼ 10648.734	99,605	96.65%	3.02%
19	134	323	139.816 ∼ 6370.372	100,680	96.80%	2.94%
20	155	412	136.496 ∼ 10318.904	132,240	96.82%	2.93%
21	120	440	109.428 ∼ 5384.872	135,617	96.75%	3.02%
22	107	281	157.476 ∼ 6367.688	88,915	97.05%	2.74%
23	129	333	116.598 ∼ 12235.022	100,831	96.67%	3.01%
24	115	354	168.565 ∼ 7962.023	139,305	96.96%	2.83%
25	85	221	101.466 ∼ 5820.621	82,900	97.08%	2.66%
26	145	410	137.351 ∼ 6965.640	122,509	96.78%	2.93%
27	97	206	119.851 ∼ 6630.647	64,598	97.02%	2.75%
28	124	357	120.403 ∼ 8296.187	111,733	96.79%	2.96%
29	114	348	123.803 ∼ 6932.012	123,985	96.64%	3.13%

*Notes* Chr, chromosome

**Fig. 7:**
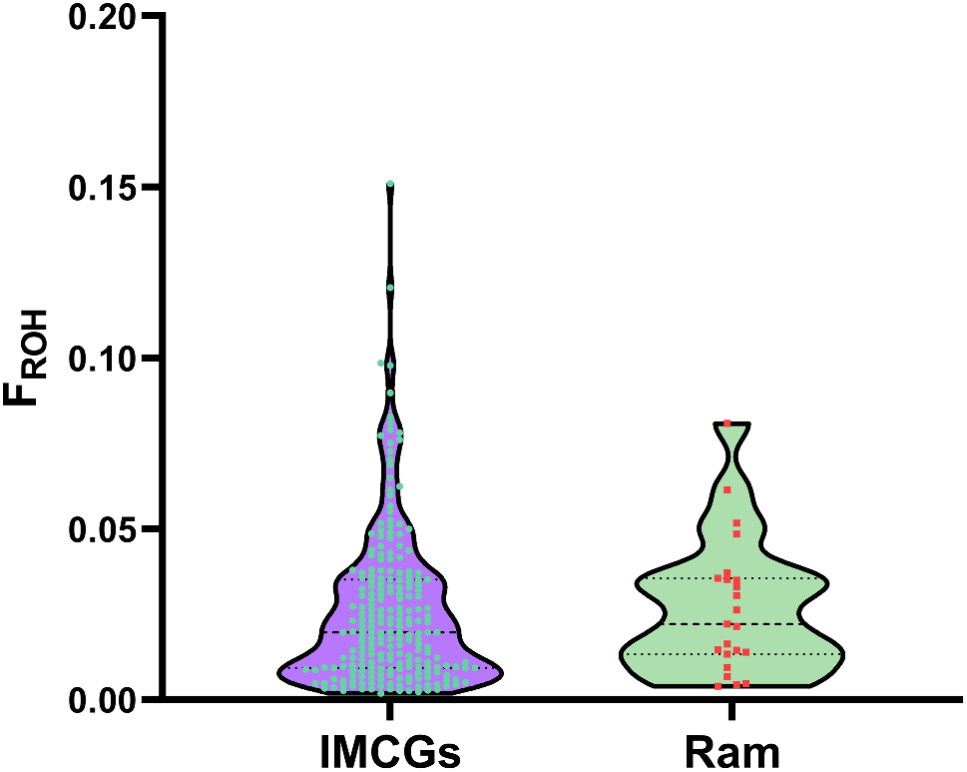
The average inbreeding coefficient derived from ROH of IMCG and ram populations

This observation may be associated with the lengths of the chromosomes in all 225 sampled IMCGs. 99.9% of the detected ROHs were smaller than 6 Mb across all IMCG populations. The abundance of short ROH suggests that recent levels of inbreeding are low. The average inbreeding coefficient derived from ROH (F_ROH_) across all the IMCG populations was 0.026 (± 0.023) (Fig. 7). However, the average inbreeding coefficient of rams was 0.027 (± 0.020), indicating that inbreeding has accumulated in ram populations. Additionally, several outliers with higher FROH values were observed among IMCG populations, indicating that some individuals are substantially inbred.

## Discussion

IMCGs are an outstanding local goat breed in China, originating from an ancient Asian lineage. They have been meticulously selected and bred over time to produce a robust, locally adapted variety with distinct characteristics, classified into three main categories (Erlangshan subtype, Albas subtype, and Alashan subtype) [1, 24]. These goats have undergone systematic improvement programs and cross-breeding efforts since the 1960s, culminating in their formal designation as “Inner Mongolia Cashmere Goats” in 1988. While many studies have concentrated on enhancing economic traits, screening differentially expressed, and molecular regulatory mechanisms of hair follicle genesis, and development and construction of regulatory networks [7, 25], the preservation of the IMCGs faces challenges, including a lack of producer engagement and reliance on conventional conservation practices. These issues have led to variability in breed quality, degradation, and inconsistency in both meat and cashmere quality, hampering the advancement of the IMCGs brand and industry. Initial conservation efforts relied heavily on government-supported activities, including specialized breeding farms, in situ conservation, and the use of genealogies for estimating genetic diversity [26, 27]. Implementing individual-based breeding strategies showed promise, further enhanced by biotechnological innovations like embryo engineering, cryopreservation of sperm, embryos, and oocytes [28, 29], and the emergence of molecular markers including AFLP, RAPD, SSR [30, 31], and more recently, SNP chips, GBS, and WGRS [18–20, 32]. These technological advancements have accelerated genetic diversity assessments for IMCGs, equipping conservationists with an advanced set of tools to precisely manage and conserve the genetic legacy of IMCGs, thereby securing the breed’s long-term survival and vitality. In our present study, we used WGRS technology to analyze 225 IMCGs sourced from a conservation farm. From this analysis, we identified 12,700,178 high-quality SNPs, which were then utilized to assess the genetic diversity, kinship, inbreeding level, and family structure within the conservation population. Our findings established a scientific foundation for the development of a conservation program tailored for the IMCG conservation population.

Genetic diversity reflects the adaptability and viability of a species or population throughout evolution. A higher genetic diversity within a population indicates a more adaptable and viable population [33]. Heterozygosity is a vital indicator of genetic variation, and a higher heterozygosity in a population signifies a richer genetic diversity [34]. The level of heterozygosity profoundly impacts an organism’s functional performance, and a positive correlation has been established between heterozygosity and fitness traits such as growth rate, survival, or fecundity [35]. In this study, the average Ho and He were 0.355 and 0.351, respectively. These findings that the population may have incorporated lineages from foreign families, necessitating further purification [36]. Islam et al. [37] used a Goat 50K SNP chip to determine that the Ho of 98 male goat breeds, including 17 Arbas cashmere male goats ranged from 0.367 to 0.401. Among these, the Ho of 17 Arbas cashmere male goats was 0.367. Genetic parameters such as MAF, PIC, SHI, Ne, and Fi are also crucial indicators for assessing the genetic diversity of populations [11, 12]. PIC values were primarily concentrated between 0.35 and 0.40 on the chromosomes, averaging 0.284. Based on the classification of PIC values where PIC < 0.25 indicates low polymorphism and 0.25 < PIC < 0.50 indicates moderate polymorphism, the IMCGs exhibited moderate genetic polymorphism [38]. The distribution pattern of SHI across various chromosomes resembled the PIC distribution, primarily concentrated between 0.65 and 0.70, with an average of 0.530. Similarly, the distribution of Ne across different chromosomes revealed a pattern of “concentrated distribution at the ends and sparse distribution in the middle”, ranging from 1.220 to 2.004, with an average of 1.582. The Fi values ranged from − 0.347 to 0.431, averaging − 0.013, and were concentrated between 0.1 and 0.1 on chromosomes. The genetic diversity results indicated moderate polymorphism in the IMCGs.

Genetic distance represents the degree of genetic variation between individuals or populations, forming the basis for constructing phylogenetic trees and analyzing population relationships [39]. The accuracy of kinship analysis is significantly influenced by the number of SNPs. Specifically, the G matrix, derived from a large number of SNPs, provides a more accurate representation of population kinship compared to the A matrix [40, 41]. This study leveraged resequencing data encompassing 1,270,078 SNPs to analyze the IBS genetic distance matrix and the G kinship matrix of the IMCG conservation population. The IBS distance was 0.283, with the rams exhibiting a slightly closer IBS distance of 0.280. This observation may suggest the presence of inbreeding among certain individuals within the population. Furthermore, the G relationship matrix revealed that 4.3% of the individuals exhibited kinship coefficients exceeding 0.1, indicating a potential risk of inbreeding within the IMCG conservation population. The results of both the G relationship matrix and the IBS distance matrix were concordant, indicating that the majority of individuals were genetically distant and genetically related to each other, with low inbreeding coefficients. However, the analysis of family structure highlighted concerns, revealing only one ram surviving in the No. 2 and No. 3 family lines. This finding is reminiscent of similar observations reported in the Liangshan pig population [34] and the Zhongwei male goat and Arbas cashmere male goat conservation group [37]. Therefore, it is imperative to closely monitor the number of rams in each family line to mitigate the risk of bloodline loss in subsequent conservation efforts.

The calculation of genomic inbreeding coefficients using ROH can be utilized to assess inbreeding in a species or population. A significant advantage of estimating these genomic inbreeding coefficients lies in the availability of chromosomal inbreeding coefficients [42]. Long ROHs reflect recent generations of inbreeding, while short ROHs indicate more distant generations of inbreeding. This is because the shorter the number of generations, the less likely it is that the ROH segments will be interrupted by recombination [43]. This coefficient is calculated as the ratio of the total length of ROH segments in the genome to the total length of the genome [44, 45]. The genomic inbreeding coefficient F_ROH_ and the lineage inbreeding coefficient FPED are moderately or strongly correlated. Notably, F_ROH_, which is calculated based on ROH, provides the closest approximation to the true inbreeding coefficient [46]. In this study, a total of 14,109 ROHs were identified among 225 individuals, with an average length ROH of 1,014.547 kb. The average inbreeding coefficient derived from ROH was 0.026, indicating a low level of inbreeding in the conserved population. While for 23 rams, it was slightly higher 0.027, inbreeding accumulation exists in rams. As the number of generations increases within the conserved population, coupled with limitations in population size and closed breeding practices, the inbreeding coefficient is inevitably to increase gradually [34]. In the conservation breeding of IMCGs, particularly the Erlangshan subtype, effectively managing inbreeding risk is critical to preserving genetic diversity and securing the population’s long-term health. This necessitates meticulous monitoring of genealogical records, strict enforcement of breeding bans between immediate relatives, and the use of advanced genetic testing to identify detrimental alleles and evaluate genetic diversity comprehensively. Collaborative relationships among IMCG breeding farms facilitate a strategic exchange of germplasm resources, while a mating rotation mechanism optimizes the gene pool by preventing pedigree over-concentration and enhancing diversity without compromising breed characteristics [47]. Simultaneously, the city of Bayannur confronts environmental challenges in its role as a significant habitat for these goats, given its semi-arid climate and fragile grasslands threatened by overgrazing [48]. Implementing rotational grazing zones that synchronize with grass growth cycles not only accelerates ecosystem recovery but also sustains biodiversity and enhances ecological resilience [49]. Coupled with scientific management practices such as adjusting livestock densities based on grassland capacities, selecting resilient and productive breeds, and providing supplementary feeding when necessary, these strategies promote a balanced and sustainable coexistence of animal husbandry and grassland health. Rainwater harvesting and water conservation further reinforce these sustainable livestock farming practices [50].

Broader conservation efforts involve the establishment of a tiered framework integrating in-situ conservation and a national genetic resource database for real-time monitoring [51]. Structured breeding programs using estimated breeding values from BLUP analysis, in conjunction with genetic testing, aim to harmonize conservation with the enhancement of economically valuable traits [52]. Furthermore, a comprehensive genetic resource bank safeguards against genetic erosion, and GWAS aids in identifying key trait markers for marker-assisted selection. Swift commercialization of improved breeds, brand cultivation, and diversified product lines cater to market demand while fostering a benefit-sharing mechanism that reinforces the synergy between conservation activities and their productive outcomes. Altogether, these integrated strategies form a robust, multifaceted approach to address the complexities of conservation breeding in the face of environmental pressures and the need for sustainable development.

## Conclusions

In summary, the conservation population of IMCGs displayed moderate polymorphism. The majority of individuals within this conserved population are genetically distant from each other, with only a few exhibiting close kinship. Nevertheless, it is necessary to prevent the loss of bloodline to ensure the continuation of IMCGs’ germplasm resources.

## Materials and methods

### Sample collection and whole genome re-sequencing

**Fig. 8:**
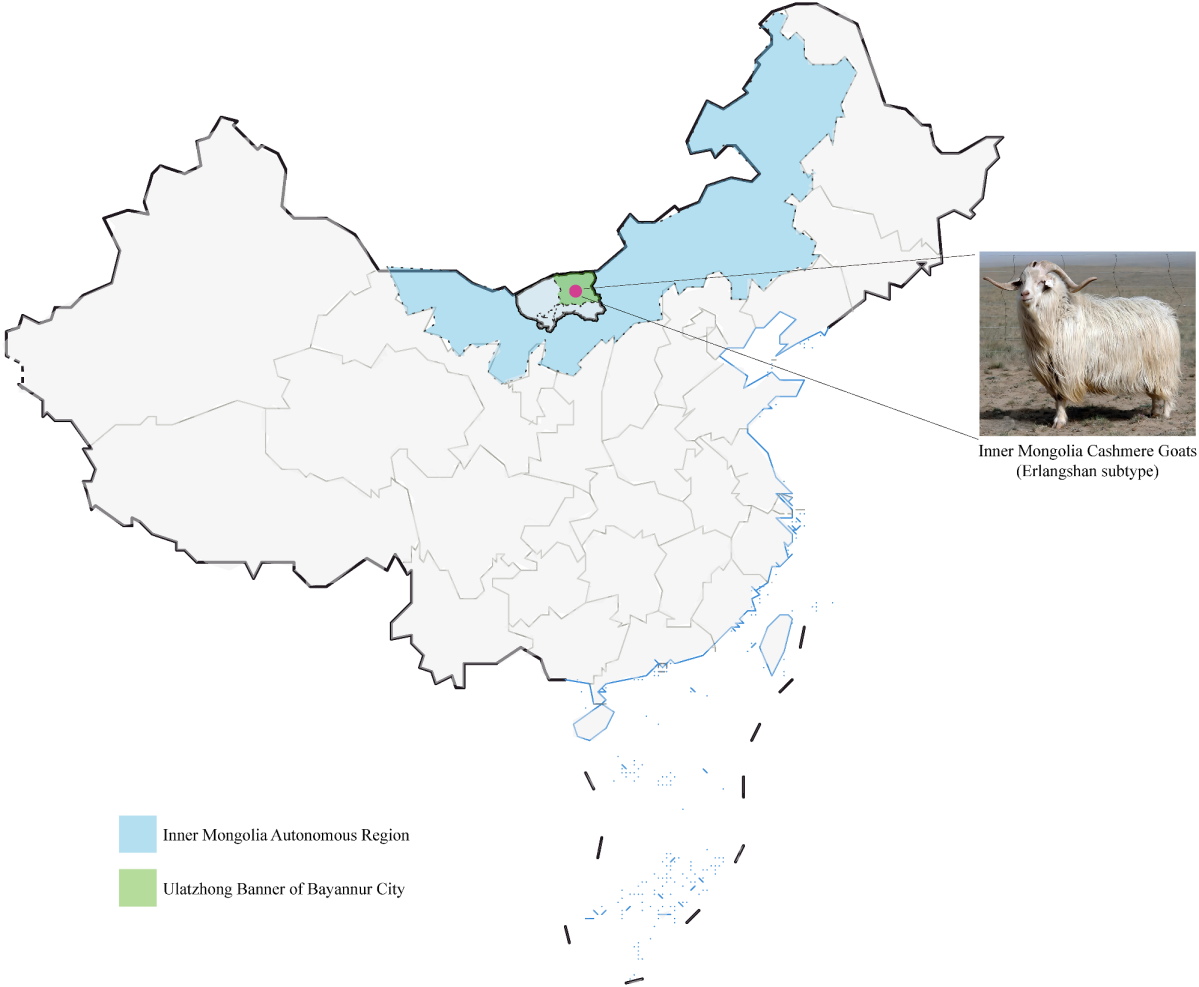
Collection site of Inner Mongolia cashmere goat tissue samples

The Erlangshan pasture (latitude 41°49′N and longitude 108°56′E) is operated by Inner Mongolia Northpeace Textile Co., Ltd., and is a national-level germplasm resource protection pasture. The pasture’s goat flock is composed entirely of purebred IMCGs, which are subdivided into ten separate family branches based on detailed genealogical records. In order to analyze the genetic diversity of the population in a comprehensive and unbiased manner, we randomly selected a representative core of individuals from each pedigree. We selected 23 active primary breeding rams and 202 ewes and their progeny, for a total of 225 samples, all of which passed stringent quality control and met the high standards required for high-throughput genome sequencing analyses, ensuring the reliability and accuracy of the study data. The geographic location of tissue sample collection for IMCG is depicted in Fig. 8. From each individual, a small piece of ear tissue (0.5 cm^2^) was excised and preserved in liquid nitrogen for subsequent DNA extraction. Genomic DNA was isolated using the standard phenol-chloroform extraction method [53]. The concentration and purity of the extracted DNA were measured through the 260/280 nm absorbance ratio using the Nanodrop 2000 spectrophotometer (Thermo Fisher Scientific Inc., Waltham, MA, USA). The qualified DNA samples obtained were stored at -80 ℃ for future utilization. For WGRS, 0.2 µg of genomic DNA was utilized to construct a sequencing library with an insert size ranging from 300 to 350 bp. Paired-end sequencing libraries (PE150) were generated and sequenced on the MGISEQ-2000 X Twenty platform (BGI, CHINA) by MolBreeding Biotech Co., Ltd., China.

### Sequencing reads quality control, reads mapping, and SNPs calling

The raw image data obtained from sequencing underwent base calling, the resulting raw data (raw reads) contained sequencing adapter, low-quality bases, and undetected bases. These adapters and low-quality bases were subsequently filtered out from each raw sequence read using Fastp (v0.20.0) software [54]. The clean reads were mapped to the goat reference genome (ARS1, GCF_001704415.1) using BWA (v0.7.17) software, which generated a sam file [55]. These SAM files were further converted to the BAM files utilizing SAM tools software (v1.90) [56]. After removed duplicates using GATK (v.1.90) software, sequencing alignment, situation coverage, and sequencing depth were analyzed using Qualimap software [57] and SAM tools software [56]. The identification of SNPs was conducted using GATK software [58], resulting in the generation of a VCF file. Strict quality control measures were implemented to eliminate unreliable genotypes using Plink (v1.90) software [59]. Individuals with call rate (CR) ≤ 90%, minor allele frequency (MAF) < 0.1, and Hardy-Weinberg equilibrium test (HWE) with a *p*-value ≤ 10^–6^ were excluded [23, 60]. Post-quality control, and high-quality SNP loci were obtained and utilized for further analysis.

### Genetic diversity, kinship, and family structure analysis

After SNPs calling and obtaining the SNP call set, we performed genetic diversity analysis to investigate patterns of genetic variation. The Plink (v.1.90) software [61] was used in conjunction with R (v.4.2.1) software to calculate genomic diversity parameters, including MAF, PIC, SHI, Ne, Fi, He, and Ho. To quantify the similarity between individuals to analyze genetic relationships and population structure, the pairwise identity by state (IBS) genetic distance matrix was calculated using Plink (v.1.90) with the parameter “-distance 1-ibs”. Additionally, the genomic (G) relationship matrix was calculated to analyze the kinship between individuals through the genome-wide SNP markers using GCTA (v.1.94.1) software [62, 63]. Based on the genomic (G) relationship matrix and the IBS distance matrix, systematic neighbor-joining phylogenetic (NJ) trees were constructed using MEGA (v10.0) [64, 65]. The final results were enhanced visually using the ITOL online tool (https://itol.embl.de/itli.cgi), providing a clearer understanding of the genetic relationships within the population.

### Detection of runs of Homozygosity and genomic inbreeding coefficient

To assess the level of inbreeding for each animal, the genomic inbreeding coefficient was determined by analyzing the number and the size of haplotype autozygosity within the genomic regions known as runs of homozygosity (ROHs). ROHs are uninterrupted stretches of homozygous genotypes commonly observed among individuals within a population, providing a reliable metric for estimating inbreeding levels. The PLINK (v1.90) software was employed to identify ROH using a sliding window of 30 SNPs across the genomes, allowing for one missing SNP and one heterozygous site within each window. A minimum gap of 100 kb was set between adjacent ROHs, and the minimum length of an ROH fragment was set to 100 kb. Subsequently, the genomic inbreeding coefficient for each animal within the conserved population was calculated based on ROHs [66].

### Electronic supplementary material

Below is the link to the electronic supplementary material.

Supplementary Material 1

## Data Availability

The datasets generated during and analyzed during the current study are available in the article. The data that support the findings of this study are available from College of Animal Science, Inner Mongolia Agricultural University, Hohhot 010018, China (Whole genome re-sequence data) and Key Laboratory of Mutton Sheep Genetics and Breeding, Ministry of Agriculture, Hohhot 010018, China (High-density SNP array data). Restrictions apply to the availability of these data, which were used under license for this study. Data are available from the authors with the permission of the Key Laboratory of Mutton Sheep Genetics and Breeding, Ministry of Agriculture, and Inner Mongolia Agricultural University.

## Abbreviations

Fi: Fix-index
G matrix: Genomic relationship matrix
GBS: Genotyping by sequencing
He: Expected heterozygosity
Ho: Observed heterozygosity
HWE: Hardy-Weinberg equilibrium
IBS matrix: Identity by state genetic distance matrix
IMCGs: Inner Mongolia cashmere goats
MAF: Minor allele frequency
NE: Effective number of alleles
PIC: Polymorphic information content
QC: Quality control
ROH: Runs of homozygosity
SHI: Shan-non-information index
SNP: Single nucleotide polymorphism
WGS: Whole genome sequencing
WGRS: Whole genome re-sequencing
