# Supplementary material for: Genetic diversity analysis of Inner Mongolia cashmere goats (Erlangshan subtype) based on whole genome re-sequencing
Source: BMC Genomics. 2024 Jul 16;25:698. doi: 10.1186/s12864-024-10485-x (PMC11253418; doi:10.1186/s12864-024-10485-x)
Supplement: Supplementary file 1 — Supplementary Material 1 [file 12864_2024_10485_MOESM1_ESM.docx]

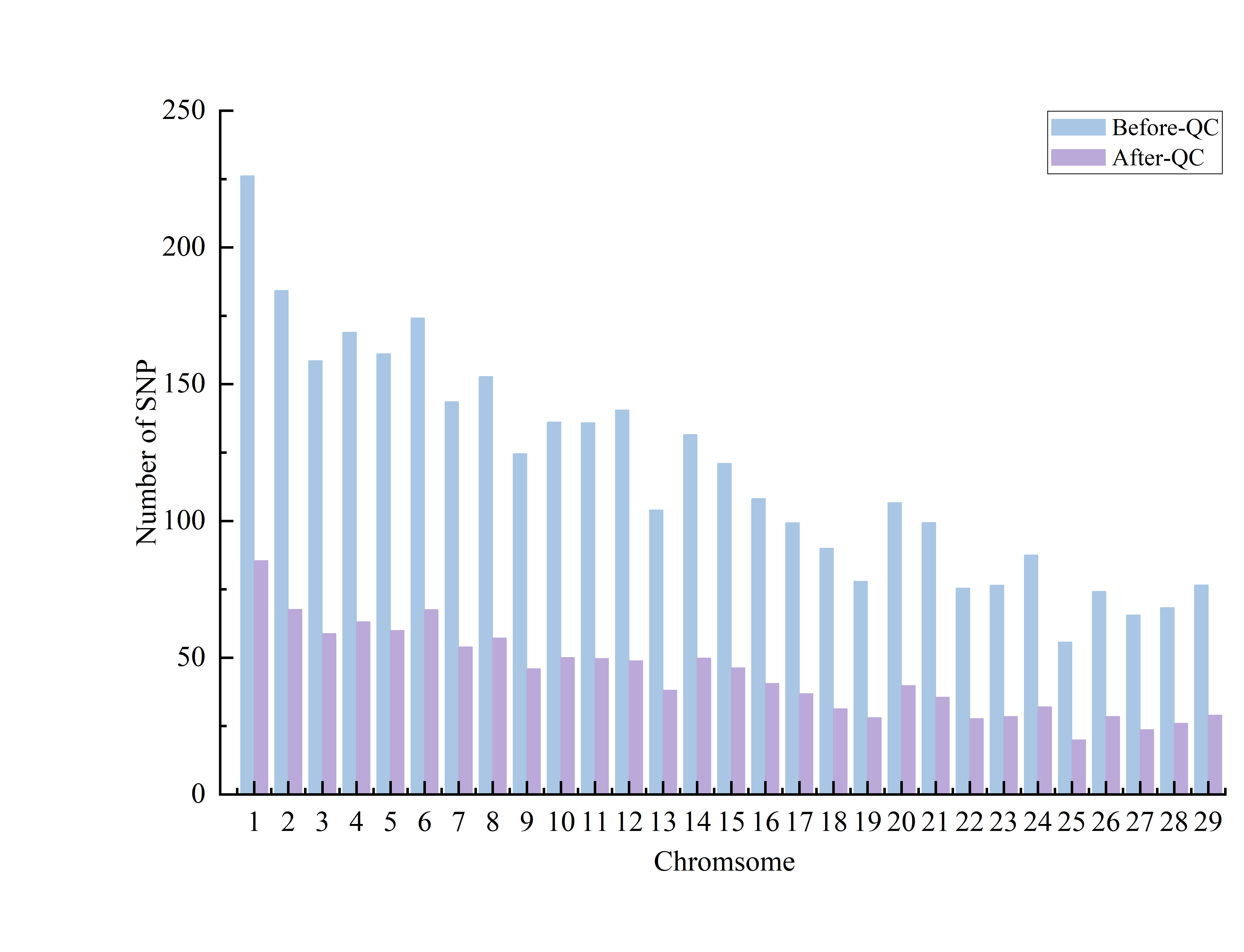


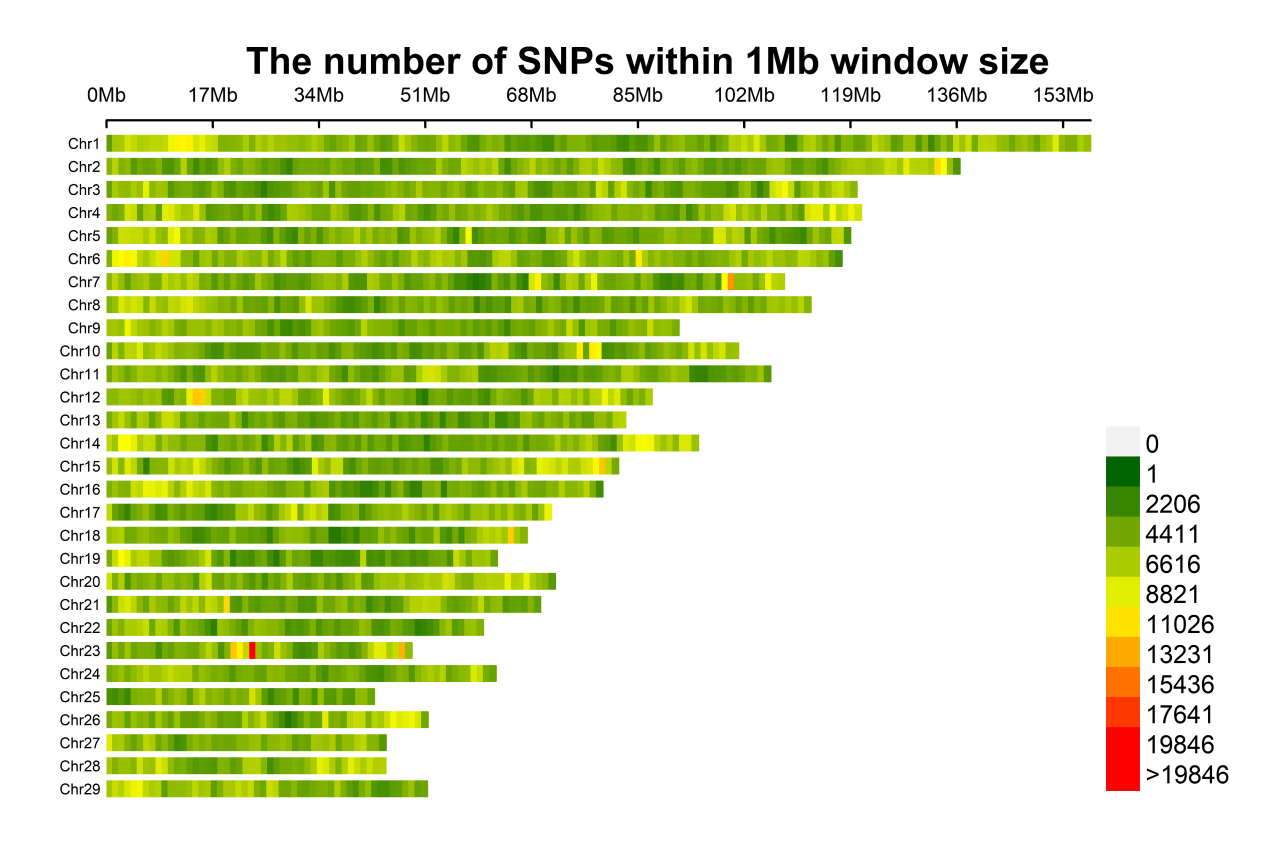
**Fig. S1** The number of SNPs on each chromosome in the genome.

### Fig. S2 The number of SNPs within 1 Mb window size. The figure shows the difference of SNP distribution in different regions of each chromosome. The color of the right legend of the figure indicates that the green area represents one SNP per 1 MB interval, while the red area denotes the presence of more than 19846 SNPs in that particular region.
